# International nurse recruitment and NHS vacancies: a cross-sectional analysis

**DOI:** 10.1186/1744-8603-1-7

**Published:** 2005-04-22

**Authors:** Amber S Batata

**Affiliations:** 1 Judge Institute of Management, Cambridge University, Trumpington Street, Cambridge CB2 1AG, UK

## Abstract

**Background:**

Foreign-trained nurse recruits exceeded the number of new British-trained recruits on the UK nurse register for the first time in 2001. As the nursing shortage continues, health care service providers rely increasingly on overseas nurses to fill the void. Which areas benefit the most? And where would the NHS be without them?

**Methods:**

Using cross-sectional data from the 2004 Nursing and Midwifery Council register, nurse resident postcodes are mapped to Strategic Health Authorities to see where foreign recruits locate and how they affect nurse shortages throughout the UK.

**Results:**

Areas with the highest vacancy rates also have the highest representation of foreign recruits, with 24% of foreign-trained nurses in the UK residing in the London area and another 16% in the SouthEast (comparable numbers for British-trained nurses are 11% and 13%, respectively). Without foreign recruitment, vacancy rates could be up to five times higher (three times higher if only Filipino recruits remained).

**Conclusion:**

The UK heavily relies on foreign recruitment to fill vacancies, without which the staffing crisis would be far worse, particularly in high vacancy areas.

## Background

The National Health Service (NHS) has been suffering the effects of a nursing shortage for the past decade as fewer women train or remain in the nurse workforce, favoring improved job market opportunities in other sectors. The same is true of many other industrialised nations. Over the next 5–10 years, the nurse shortfall is predicted to be 275,000 in the US; 53,000 in the UK and 40,000 in Australia by 2010 [1]. By 2020, the US shortfall may be as high as 800,000 [2]. Efforts to address the shortage include return-to-work initiatives, improved pay, better working environment and flexible hours, and attracting more students to nurse training programs. Even so, the negative aspects of a nursing career discourage many people from training or remaining in the nurse workforce. Perceptions of the NHS as a poor employer are particularly acute and wages remain below other professions, even other jobs within the public sector [3,4].

In this age of globalisation, many countries have turned to overseas recruitment to fill the vacancies caused by a limited or unwilling locally trained workforce. Foreign-trained nurses accounted for 23% of the nurse workforce in New Zealand in 2002; 6% in Canada (2001); 8% in Ireland (2002) and the UK (2001); and 4% in the US in 2000 [1]. And these numbers are likely to grow if domestic hiring continues to lag. In fact, in 2001, more than half of newly registered nurses were trained overseas [5]. Even doctors and pharmacists are being recruited from overseas [6,7]. But foreign recruitment comes at a price, particularly for the source countries.

The broadsheets are increasingly reporting on the 'global nursing crisis,' the 'healthcare brain drain' from developing countries and the effects the 'nurse exodus' has on quality of care in poor countries. This is especially true of African nations which not only subsidise countries like England (by investing in the training of healthcare professionals who move to industrialised nations), but suffer from growing nursing shortages of their own, exacerbating problems of existing staff shortages, high infant and maternal mortality rates, the HIV epidemic, poor nutrition and a host of other public health concerns [8-10]. As one of the world's largest importers of healthcare professionals from developing countries, the UK is in need of adopting and enforcing standards of ethical conduct in recruitment.

In 1999, the Department of Health issued guidelines on international recruitment intended to curtail poaching from poor countries already suffering from healthcare staffing shortages, such as South Africa and the Caribbean. Unless foreigners provide unsolicited applications or their governments have established programmes for professional development with the UK, the NHS was advised to avoid recruiting [11]. The guidelines were updated in 2001 to include recruitment agencies working for the NHS, but the guidelines do not apply to the independent sector and are not monitored or enforced [12]. Still, the recommendations help explain the strong presence of Filipino nurses who are explicitly encouraged to train overseas (and send income home) as part of the 2001–2004 Medium Term Philippines Development plan. Even the Philippines, however, is starting to feel the crunch of a nursing shortage and trying to find ways of ensuring that sufficient numbers of nurses, particularly nurse educators, are trained and retained in the future [1,13]. As conditions worsen in source countries, many believe that it is neither realistic nor ethical for developed nations to continue relying on foreign recruitment from disadvantaged nations [8,12,14-16].

If overseas recruitment becomes more difficult, it will have a detrimental effect on vacancy rates in the UK in future, at least in the short-term. (In the long run, perhaps staff shortages even more extreme than today could provide the impetus for major changes that would attract British-trained nurses into the profession. The changing wage structure under Agenda for Change may also help, but it is far too soon to know.) In order to understand that future effect, it helps to study the current and historic impact of overseas recruitment on the NHS. Using data from the Nursing and Midwifery Council (NMC) register from April, 2004, this paper identifies where nurses reside, by country of origin, to see how foreign recruitment affects different Strategic Health Authorities (SHAs) across England. Hypothetical vacancy rates for 2004 are estimated (based on a worst case scenario) as if the UK had no overseas recruits to determine what the shortage would look like across the country if the UK could rely only on its own stock of nurses. Though hypothetical, these numbers help shed light on the extent to which the British workforce has been unwilling to enter or remain in the nursing profession, leading to reliance on foreign sources of labour.

## 1 Methods & Data

The Nursing and Midwifery Council (NMC) maintains the register of qualified nurses, midwives and health visitors for the UK. Because any qualified nurse wishing to work in the UK must register with the NMC, it represents the entire pool of potential nurses that could be recruited into the NHS (overseas residents or retired nurses still registered notwithstanding). The NMC register is updated on a daily basis, containing over 600,000 records. An extract of data from mid-April, 2004, was provided by the NMC, containing counts of all registered nurses by 5-digit postcode by country of qualificiation (where known). While there is certainly error in assigning postcode data to areas within England, the method produces aggregate numbers comparable to the NMC's own (unpublished) analysis by reported country of residence. The NMC estimated that, as of the end of March, 2004, roughly 632,000 (or 96%) of the 660,215 registrants resided in the UK, of whom roughly 509,000 were in England, 64,000 in Scotland, 32,000 in Wales and 22,000 in Northern Ireland (with an additional 4,000 in the UK unspecified). These are roughly comparable with the counts from April, 2004, presented here (Table 1). They found almost 29,000 registrants either reside overseas or did not provide resident country or postcode, which is comparable to the estimated 31,000 unknown or foreign addresses found here and to previous estimates that roughly 5% of the register reside overseas [17]. These numbers are similar to a report on the 2002–2003 data [18]. Because the register is updated on a daily basis, published numbers will not match the current analysis exactly.

**Table 1:** Nurse staffing, registration and vacancies by SHA, 2004^*a*^

	**NHS Staffing**		**NMC Register**		**Vacancy**	
	
SHA name	wte	headcount	register	%NHS^*b*^	%vac	#vac
Norfolk, Suffolk & ambridgeshire	12312	15936	21775	73	2.3	296
Bedfordshire & Hertfordshire	6818	8684	15416	56	6.4	441
Essex	6858	8637	13976	62	3.3	230
**London**						
North West London	12627	15556	16785	93	6.6	815
North Central London	10501	12553	11379	110^*c*^	5.7	592
North East London	9416	11646	13632	85	3.8	332
South East London	11024	13930	16384	85	7.3	811
South West London	7503	9679	14437	67	2.3	170
Northumberland, Tyne & Wear	10365	12455	14588	85	1.1	113
County Durham & Tees Valley	7275	8536	12147	70	1.4	103
NE Yorkshire & N Lincolnshire	7951	9845	17842	55	2.1	174
West Yorkshire	14233	17926	20594	87	1.6	221
Cumbria & ancashire	11490	14226	21246	67	1.3	155
Greater Manchester	17422	20674	25279	82	1.6	289
Cheshire & Merseyside	16276	19730	26948	73	1.4	225
**The Southeast**						
Thames Valley	10621	13975	20044	70	2.9	295
Hampshire & Isle of Wight	9424	12564	18370	68	3.6	331
Kent & Medway	7261	9303	14562	64	2.7	195
Surrey & Sussex	12977	17258	28048	62	4.3	547
Avon, Gloucestershire & Wiltshire	12699	16836	23765	71	1.8	220
South West Peninsula	8888	10958	16902	65	0.9	77
Dorset & Somerset	6202	7945	12662	63	0.4	23
South Yorkshire	9138	10783	13355	81	0.9	85
Trent	14050	17333	26817	65	0.8	120
Leics, Northamptonshire & Rutland	7272	9041	15028	60	2.1	167
Shropshire & Staffordshire	7985	9862	16108	61	1.1	89
Birmingham & the Black Country	15288	18343	19620	94	1.9	296
West Midlands South^*d*^	7460	9793	15813	62	1.2	86
						
All London SHAs	51071	63364	72617	87	5.1	2719
Southeast Region^*e*^	40283	53100	81024	66	3.3	1368
Rest of England	199982	247543	349881	71	1.7	3421
**England TOTAL**	291336	364007	503522	73	2.6	7508
Wales	26300	-	33281	-	2.1	564
Scotland	39037	41270	66817	6.2	1.1	486
Northern Ireland	-	-	21645	-	-	-

^*a*^Nurse staff based on the NHS Workforce Census conducted in September, 2003 by SHA of work (excluding staff of special health authorities or other statutory bodies besides SHAs) [31]. Registered nurse population based on April, 2004 NMC data by postcode of residence mapped to SHA. Vacancy data from the 2004 Vacancy Survey for England [21], ISD Scotland [32] and the Statistical Directorate of the National Assembly for Wales [33]. All figures relate to qualified nurses, midwives and health visitors.

^*b*^NHS headcount divided by # registered nurses.

^*c*^Over 100% because fewer nurses reside in North Central London than work there (due to nurses who commute from other SHAs).

^*d*^Formerly named the Coventry, Warwickshire, Herefordshire & Worcestershire SHA.

^*e*^Includes SHAs of Thames Valley, Hampshire & Isle of Wight, Kent & Medway, and Surrey & Sussex.

To verify country of training, the NMC data was compared with a publication on London-based nurses. A 2002 analysis by Buchan, Finlayson and Gough found that roughly 12% of nurses reporting a London postcode were from overseas, similar to the 10% found here [19]. The lower count may be attributable to one of several reasons. First, a small number of registrants may have been misassigned to a country, or allocated to 'unknown postcode,' due to partial or inaccurate postcode reporting (which is the only geographic identifier available in this analysis). These cases would not have been assigned to a London Strategic Health Authority. Furthermore, newly registered foreign recruits may list their recruitment agency address initially, introducing error in SHA assignment for newly registered immigrants (though this should disappear upon reregistration). Finally, in previous work done by Buchan analyzing the NMC data, all foreign recruits appeared to enter the UK after 1995, despite known foreign recruitment before then. The loss of data on country of training may be attributable to a change in computer systems used by the NMC to track nurse registrations. But whatever the explanation, it appears the NMC data undercount foreign-trained nurses, excluding those registering prior to 1996 and perhaps undercounting since then as well. So the current analysis focuses on recent recruitment, subject to the limitations of the NMC data itself. (Table 2 shows that of the 67,176 new non-UK admissions to the NMC register since 1993, only 7335, or 11%, registered prior to 1996. Some of them may not have entered the UK or could have left by now. But to the extent they remain in the UK, the majority of foreign recruits will have been captured and properly attributed to their source country in the 2004 data.)

**Table 2:** Newly registered nurses in the UK, 1993–2002^*a*^

Year	UK admissions	Non-UK admissions	UK as% of all admissions
1990/91	18980	-	-
1991/92	18269	-	-
1992/93	18064	-	-
1993/94	17948	2121	89
1994/95	17411	2452	88
1995/96	16870	2762	86
1996/97	14210	3774	79
1997/98	12082	4300	74
1998/99	12974	4891	72
1999/00	14035	7383	65
2000/01	15433	9709	61
2001/02	14538	16155	47
2002/03	18048	13629	57

^*a*^Counts of newly registered trained nurses and midwives on the NMC register (formerly the UKCC register) [17]. Data from 1990–1992 only available for UK admissions [34]. The year represents all nurses newly registered between April 1 of that year, and 31 March of the following year.

Postcode data was mapped to SHA code using the XYZ Digital Map Company's PostZon file, mapping 7-digit postcodes to SHAs (based on data supplied by Royal Mail) [20]. For NMC postcodes with multiple SHAs (particularly where only the first two postcode digits were reported in the register), nurse counts were split across SHAs according to the proportion of 7-digit postcodes within the NMC postcode assigned to an SHA.

The Department of Health Vacancy Survey began in 1999 to the present, collecting data on vacancy rates in England by occupation code by Trust and Health Authority [21]. The survey asks respondents to report total number of positions that remained vacant for at least three months as of 31 March of each year. The vacancy rate is calculated as the number of openings that have remained vacant for 3 months or more, divided by the number of whole-time equivalent (wte) staff-in-post + number of vacancies.

Using cross-sectional data from the NMC and Department of Health for Spring, 2004, hypothetical vacancy rates are calculated assuming all posts held by foreign recruits remained vacant. In other words, if the NHS had not been able to address labour shortages using foreign sources, how much worse might the vacancy problem be today? Assume:

1. All overseas recruits work full-time within their SHA for the NHS (therefore, #UK-trained NHS wte staff = #NHS wte - #foreign-trained within the SHA);

2. There were no foreign-trained nurses working in the UK, and;

3. Wages and job characteristics would be no different today without foreign recruits than they are with (notwithstanding the added stress of higher vacancies, which would probably further increase vacancies).

Obviously these assumptions are severe and improbable, but they are needed to present a worst case scenario and to calculate the upper boundary of vacancies in the absence of foreign recruitment.

## 2 Results & Discussion

Table 1 shows staffing levels (wte and headcounts), NMC register counts, proportion of the register working in the NHS (headcount divided by register count), and vacancy data by SHA in 2004, for all qualified nurses, midwives and health visitors. The register is based on postcode of residence, rather than postcode of work, so the estimated 'proportion working in NHS' will be biased upwards by commuters coming to work in an SHA (the effect of which may be muted somewhat if resident nurses work in the private sector, commute out of the SHA or do not work, in comparable numbers to the incoming commuters). For example, in North Central London, obviously many nurses commute to and work in the SHA in addition to NHS nurses residing in the SHA boundaries, so more than 100% of resident nurses appear to work there. This commuting bias probably exists for much of the London area. It may also explain the lower proportion of registered nurses in the Southeast region who work within the Southeast SHAs if they travel to London to work (only 66% in the Southeast relative to the national average of 73% of registered nurses working in the NHS).

Without data linking nurses to their postcode of work, it is impossible to know whether the low counts in the Southeast are caused by working in the private health sector; commuting to NHS jobs in London or other SHAs; working outside health altogether; or not working. About 25% of NMC-registered nurses are known to work outside the NHS [22], but neither the registry data nor other micro-datasets allow much detailed analysis of what fraction of trained nurses in an area choose to work (at all, or for the NHS, in particular). The job choices of nurses can be studied using data of trained or training nurses in the Quarterly Labour Force Survey from 1999–2003 [23]. Of roughly 1100 qualified or qualifying nurses (in each year of the data), about 64% worked as a nurse and another 17% did not work. Of those working as a nurse, 60% worked full-time, 83% of whom worked in the public sector. While the QLFS does allow this type of calculation at the national level, the sample size is insufficient to provide reliable breakdowns by SHA (with only about 40 nurses per Authority).

Table 1 also provides aggregate vacancy rates by SHA. Obviously, vacancy rates are highest in London, which also sees a higher proportion of the registered nurse population working in the NHS (though this statistic is biased upwards by commuters from outside the London area). But these aggregate numbers mask the problem of encouraging British citizens to train and work in nursing, as vacancies are increasingly being filled by foreign-trained recruits. The next step is to determine the extent to which shortage areas rely on overseas recruitment.

Table 2 shows a steady decline in nurses joining the NMC register during the 1990s, though it has steadily grown in the past six years. And as UK admissions (i.e. newly registered nurses who trained within the UK) fell, foreign-trained admissions rose. The past ten years have seen a tremendous change in international nurse mobility. This trend is similar for newly registered doctors over the 1990s as non-UK, non-EEA sources increased from 33% of newly registered doctors in 1994 to 44% by 2002 [8]. Table 3 shows the increase in newly registered nurses in the UK from 1998–2003 by source country grouping. The top panel shows foreign recruits from the top 25 non-EU countries, and the bottom panel provides counts of all newly registered nurses. Clearly the top 25 source countries have come to dominate international recruitment, representing 70% of newly recruited nurses in 1998, but 90% by 2003. Much of this increase is attributable to recruitment from the Philippines, which accounted for only 1% of newly registered overseas recruits in 1998, but over 40% by 2000. And while South Africa and India are contributing a growing number of nurses to the UK register, recruitment from the US, Canada, New Zealand and Australia fell significantly over this period. Of the 30,800 foreign-trained recruits known to reside in the UK and on the NMC register in mid-April, 2004, almost two-thirds (19,500) are from Asia; another 7000 from Africa and 2400 from major English-speaking countries (Canada, New Zealand, Australia and the United States). Only a handful of recruits are from Europe (1500) and fewer still from Latin America (300). But overseas recruits are not uniformly distributed across the UK once they arrive.

**Table 3:** Newly registered overseas nurses^*a*^

Country	98/99	99/00	00/01	01/02	02/03
Philippines	52	1052	3396	7235	5593
South Africa	599	1460	1086	2114	1368
India	30	96	289	994	1830
Australia	1335	1209	1046	1342	920
New Zealand	527	461	393	443	282
Canada	196	130	89	79	52
USA	139	168	147	122	88
West Indies	221	425	261	248	208
Pakistan	3	13	44	207	172
Malaysia	6	52	34	33	27
Singapore	13	47	48	43	25
Nigeria	179	208	347	432	509
Zimbabwe	52	221	382	473	485
Ghana	40	74	140	195	251
Kenya	19	29	50	155	152
Zambia	15	40	88	183	133
Mauritius	6	15	41	62	59
Malawi	1	15	45	75	57
Botswana	4	0	87	100	39
Other^*b*^	0	0	0	0	131
					
Top25 Total	3437	5715	8013	14535	12381
Total foreign-trained	4891	7383	9709	16155	13629
Total UK-trained	12974	14035	15433	14538	18048
Total new registrants	17865	21418	25142	30693	31677
					
%Overseas from Philippines	1	14	35	45	41
%Overseas from Top25	70	77	83	90	91
%New registrants from overseas	27	34	39	53	43

^*a*^Counts of newly registered trained nurses and midwives provided by the Nursing and Midwifery Council based on their register of qualified nurses and midwives. Based on the top 25 non-EU foreign source countries only (top panel); middle and lower sections include counts of all newly registered nurses. Data obtained from NMC and [5].

^*b*^Includes 23 each from Poland and Sri Lanka; 22 each from the Czech Republic and Saudi Arabia; 21 from Nepal and 20 from Japan in 2002/03.

Using the NMC mid-April data, broken down by country of training by postcode, an SHA of residence is assigned based on the reported postcode sector (the 5-digit postcode, assuming a valid or partial postcode is provided and is within the UK). Table 4 presents data on the NMC register, broken down by SHA of resident postcode, by training source (foreign or UK). Scotland has the lowest number of registered foreign recruits as a fraction of all registered nurses with only 1.3%, while England has the highest at 5.5%. SHAs outside of London and the Southeast experienced average foreign representation of only 4.3% (of all registered nurses within the SHA). SHAs with high fractions of foreign recruits (5.5% or more of all registered nurses), are either in London (over 10%) and the Southeast (6.2%) or contain some of the largest cities in England (namely Manchester, Birmingham and Bristol). Leeds (in the West Yorkshire SHA) has a slightly lower foreign presence (5%) with only Liverpool and Sheffield (the Cheshire&Merseyside and South Yorkshire SHAs) among Britain's seven largest cities with very low shares of foreign-trained nurses (less than 4.5% of their potential workforce). Unsurprisingly, the SHAs with higher proportions of overseas recruits are also the ones with the highest vacancy rates (Table 1). Apparently, the worse the nursing shortage, the more active the foreign recruitment efforts (assuming high foreign representation does not drive vacancies, but vacancies drive foreign recruitment).

**Table 4:** Overseas and domestic registered nurses, April 2004^*a*^

		Training Source^*b*^				
			
SHA name	Total	UK	Intl	%UK	%Intl	%Intl in SHA^*c*^
Norfolk, Suffolk & ambridgeshire	21775	20364	1411	93.5	6.5	4.6
Bedfordshire & Hertfordshire	15416	14305	1111	92.8	7.2	3.6
Essex	13976	13200	776	94.5	5.6	2.5
**London**						
North West London	16785	14251	2534	84.9	15.1	8.2
North Central London	11379	10215	1164	89.8	10.2	3.8
North East London	13632	12330	1302	90.5	9.6	4.2
South East London	16384	15093	1291	92.1	7.9	4.2
South West London	14437	13260	1177	91.9	8.2	3.8
Northumberland, Tyne & Wear	14588	14080	508	96.5	3.5	1.7
County Durham & Tees Valley	12147	11867	280	97.7	2.3	0.9
NE Yorkshire & N Lincolnshire	17842	17496	346	98.1	1.9	1.1
West Yorkshire	20594	19569	1025	95.0	5.0	3.3
Cumbria & ancashire	21246	20647	599	97.2	2.8	1.9
Greater Manchester	25279	23882	1397	94.5	5.5	4.5
Cheshire & Merseyside	26948	25758	1190	95.6	4.4	3.9
**The Southeast**						
Thames Valley	20044	18461	1583	92.1	7.9	5.1
Hampshire & Isle of Wight	18370	17355	1015	94.5	5.5	3.3
Kent & Medway	14562	13930	632	95.7	4.3	2.1
Surrey & Sussex	28048	26274	1774	93.7	6.3	5.8
Avon, Gloucestershire & Wiltshire	23765	22178	1587	93.3	6.7	5.2
South West Peninsula	16902	16653	249	98.5	1.5	0.8
Dorset & Somerset	12662	12206	456	96.4	3.6	1.5
South Yorkshire	13355	12916	439	96.7	3.3	1.4
Trent	26817	26049	768	97.1	2.9	2.5
Leics, Northamptonshire & Rutland	15028	14546	482	96.8	3.2	1.6
Shropshire & Staffordshire	16108	15536	572	96.5	3.6	1.9
Birmingham & the Black Country	19620	18348	1272	93.5	6.5	4.1
West Midlands South	15813	15275	538	96.6	3.4	1.8
						
All London SHAs	72617	65149	7468	89.7	10.3	24.2
Southeast Region	81024	76020	5004	93.8	6.2	16.2
Rest of England	349881	334875	15006	95.7	4.3	48.7
**England TOTAL**	503522	476044	27478	94.5	5.5	89.2
Wales	33281	32022	1259	96.2	3.8	4.1
Scotland	66817	65935	882	98.7	1.3	2.9
Northern Ireland	21645	20591	1054	95.1	4.9	3.4

^*a*^Qualified nurses, midwives and health visitors registered with the NMC in mid-April, 2004, known to reside in the UK. All those residing outside the UK or with unknown postcode are excluded (31154 cases). Counts of registered nurses in the Channel Islands or Isle of Mann are included in UK total, but not assigned to any SHAs or country totals within this table. Note: International refers to registered nurses who trained outside the UK.

^*b*^The #UK(or foreign)-trained registrants divided by #registrants residing in the SHA.

^*c*^Calculated as #foreign-trained nurses in this SHA divided by total #foreign-trained nurses on the NMC register known to reside in the UK (30,808).

Of the roughly 30,000 foreign-trained nurses residing in the UK, 24% are in the London area and another 16% in the SouthEast (with 49% in the rest of England and the remaining 11% divided among Wales, Scotland and Northern Ireland). Comparable numbers for British-trained registrants living in the UK are 11% in London, 13% in the Southeast and 56% in the rest of England. While there are problems with using NMC registration address to assign the SHA of work, the data can roughly determine the distribution of nurses across England (with some error for commuters and misassigned postcodes), and provide reasonable estimates of the pool of potential nurses located within SHA boundaries.

Clearly a disproportionate share of foreign-trained nurses live (and probably work) in the London area, helping to lower London vacancy rates beyond what they would be were international recruitment not possible. Table 5 presents upper bound estimates for hypothetical vacancy rates in the absence of international recruitment. Without any foreign recruits, vacancy rates could be as high as 12% for England, but higher for individual SHAs, particularly in London where aggregate vacancies could rise above 20%. These vacancy rates are three to five times greater than current estimates. Even allowing for Filipino recruitment, vacancies would still be two to three times greater. Of course, given the assumption that all foreign-trained recruits work full-time for the NHS (rather than the independent sector that probably recruited them, for example), these numbers are an upperbound, with more reliable predicted vacancies lying somewhere between current rates and those in Table 5. And few countries need worry about the existing stock of nurses from the Philippines as they were actively encouraged by their government to work overseas. In future, however, as their domestic shortage worsens, supply may fall, or ethical considerations may prevent continued (heavy) reliance on the Philippines. But for the UK, with or without Filipino nurse recruits, it is clear the labour shortage would be far worse today in the absence of foreign labour sources, particularly (and unsurprisingly) in London and the Southeast (and the Bedfordshire & Hertfordshire SHA).

**Table 5:** Hypothetical Vacancies without Foreign Recruits ^*a*^

SHA name	%vac^*b*^	%vacancyUK^*c*^	#vacancyUK^*d*^	%vacUK_Filipino^*e*^
Norfolk, Suffolk & ambridgeshire	2.3	13.3	1707	7.4
Bedfordshire & Hertfordshire	6.4	22.5	1552	16.1
Essex	3.3	14.3	1006	9.6
**London**				
North West London	6.6	27.2	3349	20.0
North Central London	5.7	17.0	1756	14.0
North East London	3.8	18.7	1634	13.8
South East London	7.3	19.0	2102	15.1
South West London	2.3	18.6	1347	13.1
Northumberland, Tyne & Wear	1.1	5.9	621	2.7
County Durham & Tees Valley	1.4	5.2	383	2.9
NE Yorkshire & N Lincolnshire	2.1	6.4	520	5.0
West Yorkshire	1.6	9.2	1246	5.2
Cumbria & ancashire	1.3	6.4	754	4.5
Greater Manchester	1.6	9.5	1686	7.8
Cheshire & Merseyside	1.4	8.7	1415	6.2
**The Southeast**				
Thames Valley	2.9	18.5	1878	11.8
Hampshire & Isle of Wight	3.6	14.5	1346	7.6
Kent & Medway	2.7	11.3	827	7.3
Surrey & Sussex	4.3	18.3	2321	11.4
Avon, Gloucestershire & Wiltshire	1.8	14.4	1807	9.9
South West Peninsula	0.9	3.7	326	2.8
Dorset & Somerset	0.4	8.0	479	4.8
South Yorkshire	0.9	5.8	524	5.1
Trent	0.8	6.2	888	3.8
Leics, Northamptonshire & Rutland	2.1	8.2	649	6.2
Shropshire & Staffordshire	1.1	8.3	661	3.9
Birmingham & the Black Country	1.9	10.2	1568	5.9
West Midlands South	1.2	8.3	624	5.6
				
All London SHAs	5.1	18.9	10187	14.4
Southeast Region	3.3	15.3	6372	9.3
Rest of England	1.7	9.1	18427	6.0
**England TOTAL**	2.6	12.1	34986	8.3
Wales	2.1	6.8	1823	3.7
Scotland	1.1	3.1	1368	2.5

^*a*^Assumes no foreign-trained nurses ever came to the UK and that existing NHS staffing counts include full employment of foreign-trained nurses (i.e. 100% of international recruits work full-time for the NHS within their resident SHA, and nurses working in the private health sector or not working at all are attributable entirely to the domestically trained population). While subject to measurement error and strong assumptions, numbers represent upper bound on possible vacancies without foreign recruitment (assuming wages and working conditions would not have improved more over the past few years to attract more locally-trained nurses).

^*b*^Actual 3-month vacancy rate reported by Department of Health.

using wte count from March, 2004 (collected in the Vacancy Survey and used in the calculation of vacancy rates).

^*d*^#vacUK = #vacancy (regular, Table 1) + # foreign-trained nurses.

## 3 Conclusion

There is a growing literature concerning nurse labour shortages and foreign recruitment. This paper identifies where foreign recruits move in the UK and estimates that vacancy rates could be three to five times higher without such a strong foreign-trained presence. This is especially true of the highest vacancy areas, like London and the SouthEast, which could otherwise have double digit vacancy rates (excluding all international recruitment other than from the Philippines). An estimated 100,000 nurses on the NMC register were over the age of 54 and less than 12% (fewer than 66,000) are under the age of 30 [17]. This lopsided age distribution will cause further problems as the 55+ cohort retires over the next ten years. Without overseas recruits to rely on, it is not clear the domestic market can supply the necessary staff. So without major changes, the UK's reliance on foreign-trained nurses will continue (in the forseeable future), and nursing is likely to remain a safe career choice for foreigners hoping to emigrate to the UK.

Reliance on foreign recruitment (not just in the UK, but the USA and other industrialised nations) poses two important questions for policymakers and researchers. The first of course is how to mitigate the impact of nurse emigration on source countries. Developing countries cannot hope to compete with the higher salaries and better working conditions offered by the UK or other developed economies, leaving their health services (especially in rural areas) with labour shortages of their own [24]. For example, an estimated two-thirds of the Jamaican nurse population has emigrated, leaving Jamaica to fill the void from Cuba [25]. And an estimated 18,000 nurses from Zimbabwe lived overseas in 2002 [8]. Of course some migration, particularly temporary, can be beneficial to source countries as their workforce gains additional skills and experience working overseas and, possibly, sends remittance income home, but what little evidence exists seems to suggest only a small proportion of nurses or other skilled migrants return to their home countries[8,26].

Restricting emigration or taxing leavers (as was common in the 1970s) have a variety of problems and will not eliminate individual workers' desire to leave [25]. Any long run solution should involve strategies to encourage workers to stay, through better working conditions, improved wages, or other positive inducements. These efforts could have the added benefit of encouraging even more people to train as healthcare providers in developing countries. To better understand and address these problems, further research is needed to quantify the effect of nurse migration on developing countries; estimate what fraction of the nurse workforce emigrates from each country; determine what fraction of emigrating nurses remain permanently overseas or return home (and in what timeframe); and study the effect of various policy options to encourage more nurses to stay in their home countries. So far, data from source countries is limited, and even industrialised nations have little information tracking skilled workforce migrants [24].

The second question to address is how to encourage more people in industrialised nations to train as nurses. Just as developing countries have difficulty competing with developed economies in the nurse labour market, the NHS cannot effectively compete with other employers (in healthcare or other sectors) in the domestic labour market. There are several reasons for the declining number of nurses in the UK, including working conditions, low wages, the cost of living, the changing nature of the job, feeling valued and of course, outside employment opportunities. The labour supply elasticity literature from the UK suggests nurses are relatively unresponsive to wage increases, with a 10% increase in wages leading to an estimated 4% increase in hours worked or 6% increase in the probability of working [27,28]. However, survey data suggests pay does drive decisions (or intentions) to quit. And a comparison of nurses' wages with those of other nonmanual female workers in the UK suggests nurse wages increased in real terms over the past 20 years, but fell relative to other workers (presumably making other careers more attractive by comparison).

Furthermore, geographic variation in vacancy rates across the UK is partially driven by variation in housing costs. This also suggests low wages may be the culprit, since the relatively flat pay structure in the NHS does not adequately adjust wages in high cost areas (there is an adjustment, but it is small compared with London housing costs, for example), driving nurses away [29]. Another reason behind the nursing shortage in England is poor labour force planning in the early 1990s, during which the number of training posts was intentionally reduced. Since 1994, training positions have increased with the Department of Health and now, Strategic Health Authorities, setting the number of training positions needed. However, by failing to take into account demographic trends and increased competition from the private sector (in terms of rising demand from an ageing patient population, an ageing nurse workforce which will need to be replenished, and growth in private sector employment opportunities for nurses), the targets continued to fall short of demand, at least in the 1990s [30]. Ideally, future demand will be met from the domestic labour supply, but this requires better educational planning and improved working conditions and pay to train and retain appropriate numbers of nurses.

Additional research is also needed on the labour supply of nurses in industrialised nations. The UK has a good starting point as the Nursing and Midwifery Council registry provides an invaluable resource containing geographic and basic training data for all nurses. With some effort, it could be used to track migration patterns of nurses within the UK as nurses update their information every few years (when they re-register). A long-term strategy might also entail adding employment information to the database, to determine where nurses work, whether in the NHS or private sector, and how far a commute they experience depending on their SHA of employment. And any of these analyses could be broken down by source country of training, to see whether foreigners choose different career paths or locations from the domestically-trained nurse population. Coupled with vacancy and turnover rate data from the Department of Health, more detailed information about the student nurse population (particularly dropout rates and job choice following graduation), and better information about working conditions and wages across Strategic Health Authorities, this data could help the NHS better understand what policies to develop and where to target them (demographically or geographically) in order to improve nurse recruitment within the UK.

## 4 Competing interests

Financial support from Bristol-Myers Squibb is gratefully acknowledged. I have no competing interests.

## References

[B1] Aiken LH, Buchan J, Sochalski J, Nichols B, Powell M (2004). Trends in international nurse migration. Health Affairs.

[B2] Bureau of Health Professions (2002). Projected supply, demand and shortages of registered nurses: 2000–2020. http://bhpr.hrsa.gov/healthworkforce/reports/rnproject/report.htm.

[B3] Arnold J Cut to the Chase. Health Service Journal.

[B4] Finlayson B, Dixon J, Meadows S, Blair G (2002). Mind the gap: the policy response to the NHS nursing shortage. BMJl.

[B5] Buchan J, Parkin T, Sochalski J (2003). International nurse mobility: trends and policy implications. http://www.rcn.org.uk/downloads/InternationalNurseMobility-April162003.doc.

[B6] Goldacre MJ, Davidson JM, Lambert TW (2004). Country of training and ethnic origin of UK doctors: database and survey studies. British Medical Journal.

[B7] Matowe L, Duwiejua M, Norris P Is there a solution to the pharmacist brain drain from poor to rich countries?. The Pharmaceutical Journal.

[B8] Bach S (2003). International migration of health workers: Labour and social issues.

[B9] Dugger CW An exodus of African nurses puts infants and the ill in peril.

[B10] Anonymous Africa's health-care brain drain.

[B11] Department of Health (1999). Guidance on international nursing recruitment. http://www.dh.gov.uk/assetRoot/04/03/47/94/04034794.pdf.

[B12] Buchan J (2004). International rescue? The dynamics and policy implications of the international recruitment of nurses to the UK. Journal of Health Services Research & Policy.

[B13] Global Scholarship Alliance (2003). Program launched to reverse global nursing crisis. The State Employee.

[B14] Buchan J, Sochalski J (2004). The migration of nurses: trends and policies. Bulletin of the WHO.

[B15] Buchan J, Jobanputrar R, Gough P (2004). Experts don't make exports. Health Service Journal.

[B16] Anonymous (2004). Overseas recruitment: planet poaching or doing a world of good?. Health Service Journal.

[B17] Royal College of Nursing (2003). More nurses, working differently: A review of the UK nursing labour market in 2002. http://www.rcn.org.uk/publications/pdf/LabourMarketReview2002.pdf.

[B18] Nursing and Midwifery Council (2004). Statistical analysis of the register: 1 April 2002 to 31 March 2003. http://www.nmc-uk.org/nmc/main/publications/Annualstatistics2002_2003.pdf.

[B19] Buchan J, Finlayson B, Gough P (2002). In capital health? Meeting the challenges of London's health care workforce.

[B20] XYZ Digital Map Company PostZon file 2004. http://www.xyzmaps.com/postcode.htm.

[B21] Department of Health NHS Workforce Vacancy Survey 2004. http://www.publications.doh.gov.uk/public/vacancysurvey.htm.

[B22] UK Central Council (UKCC) for Nursing, Midwifery and Health Visiting (2002). The professional, educational and occupational needs of nurses and midwives working outside the NHS. http://www.nmc-uk.org/nmc/main/publications/2523NeedsPagesNHS.pdf.

[B23] Office for National Statistics Labour Market Statistics Group Quarterly Labour Force Survey, all Quarterly files December 1998 to November 2003 [computer files].

[B24] Martineau T, Decker K, Bundred P (2004). Brain drain of health professionals: from rhetoric to responsible action. Health Policy.

[B25] Lowell BL, Findlay A (2001). Migration of highly skilled persons from developing countries: impact and policy responses.

[B26] Findlay A (2001). From brain exchange to brain gain: policy implications for the UK of recent trends in skilled migration from developing countries.

[B27] Rice N (2003). The labour supply of nurses in the UK: evidence from the British Household Panel Survey. Working paper.

[B28] Skatun D, Antonazzo E, Scott A, Elliott RF (2002). Attracting qualified nurses back into nursing: an econometric analysis of labour supply. Working paper.

[B29] Batata A (2005). Presenting the evidence: why might nurses choose not to work for the NHS?. Proceedings from the 2004 Strategic Issues in Health Care Management conference Forthcoming.

[B30] National Audit Office (2001). Educating and training the future health professional workforce for England.

[B31] Department of Health, Statistics (Workforce) Division NHS hospital and community health services non-medical workforce census, England. Leeds: Department of Health.

[B32] ISD Scotland Workforce Statistics. http://www.isdscotland.org.

[B33] Statistical Directorate, National Assembly for Wales NHS staff vacancies at 31 March 2004. http://www.wales.gov.uk/keypubstatisticsforwalesheadline/content/health/2004/hdw20040825-e.htm.

[B34] Buchan J, Seccombe I, Smith G (1998). Nurses work: an analysis of the UK nursing labour market.

